# Hybrid surgical-electrophysiological epicardial cryoablation for ventricular tachycardia storm from a deep intramural substrate in hypertrophic cardiomyopathy: a case report

**DOI:** 10.1093/ehjcr/ytag810

**Published:** 2026-10-27

**Authors:** Tadashi Fujino, Shintaro Yao, Kenji Okubo, Tomohiro Mizuno, Takanori Ikeda

**Affiliations:** Department of Cardiovascular Medicine, Toho University Faculty of Medicine, 6-11-1 Omorinishi, Ota-ku, Tokyo 143-8541, Japan; Department of Cardiovascular Medicine, Toho University Faculty of Medicine, 6-11-1 Omorinishi, Ota-ku, Tokyo 143-8541, Japan; Cardiovascular Center, Yokosuka Kyosai Hospital, 1-16 Yonegahamadori, Yokosuka, Kanagawa 238-8558, Japan; Department of Cardiovascular Surgery, Toho University Faculty of Medicine, 6-11-1 Omorinishi, Ota-ku, Tokyo 143-8541, Japan; Department of Cardiovascular Medicine, Toho University Faculty of Medicine, 6-11-1 Omorinishi, Ota-ku, Tokyo 143-8541, Japan

**Keywords:** Epicardial cryoablation, Ventricular tachycardia, Hypertrophic cardiomyopathy, Deep intramural substrate, Hybrid surgical ablation, Minimally invasive cardiac surgery, Case report

## Abstract

**Background:**

Ventricular tachycardia (VT) associated with hypertrophic cardiomyopathy (HCM) may arise from deep intramural substrates and remains challenging to treat when conventional endocardial or percutaneous epicardial approaches are ineffective or infeasible.

**Case summary:**

A 63-year-old man with HCM presented with drug-resistant VT storm. Endocardial mapping showed marked narrowing of the left ventricular (LV) cavity at the midventricular level, without abnormal potentials. Pace mapping demonstrated the closest match at the distal anterolateral coronary venous branch, suggesting a deep intramural origin. A subxiphoid epicardial approach was precluded by prior oesophageal reconstruction; therefore, minimally invasive cardiac surgery via left thoracotomy enabled epicardial cryoablation using the cryoICE system. Epicardial mapping during VT revealed a low-voltage area with fractionated electrograms at the apicolateral LV. Activation time did not span the full VT cycle length, suggesting an epicardial exit with a deep intramural isthmus. VT terminated during the first cryoapplication, followed by two additional applications (total 15 min). No VT recurrence was observed during 1 year of follow-up after discontinuation of amiodarone. Postprocedural cardiac magnetic resonance demonstrated transmural enhancement at the cryoablation target site despite a myocardial thickness of approximately 15 mm, suggesting successful deep lesion formation.

**Discussion:**

This case demonstrated that hybrid surgical-electrophysiological cryoablation can provide effective treatment for VT arising from deep intramural substrates when conventional endocardial and percutaneous epicardial approaches are ineffective or infeasible. Surgical cryoablation was associated with transmural enhancement despite marked myocardial hypertrophy and resulted in durable arrhythmia suppression.

Learning points

- VT in HCM may originate from a deep intramural substrate that is inaccessible by conventional endocardial ablation.
- Previous thoracic surgery may preclude standard subxiphoid epicardial access and require alternative hybrid surgical approaches.
- Minimally invasive thoracotomy-guided epicardial cryoablation may achieve deep lesion formation and provide durable VT suppression in selected patients.

## Introduction

Hypertrophic cardiomyopathy (HCM) is associated with ventricular arrhythmias and sudden cardiac death. Although implantable cardioverter-defibrillators effectively prevent arrhythmic death, recurrent ventricular tachycardia (VT) and electrical storm remain challenging clinical problems in a subset of patients.^1^

Catheter ablation has become an important therapeutic option for drug-refractory VT. However, successful ablation may be difficult when the arrhythmogenic substrate is located within the epicardium or deep intramural myocardium, particularly in patients with marked ventricular hypertrophy.^2^

We report a patient with HCM and refractory VT storm successfully treated with minimally invasive surgical epicardial cryoablation, resulting in durable arrhythmia control, with post-procedural cardiac magnetic resonance (CMR) imaging demonstrating transmural enhancement at the treated site.

## Summary figure

**Figure:**
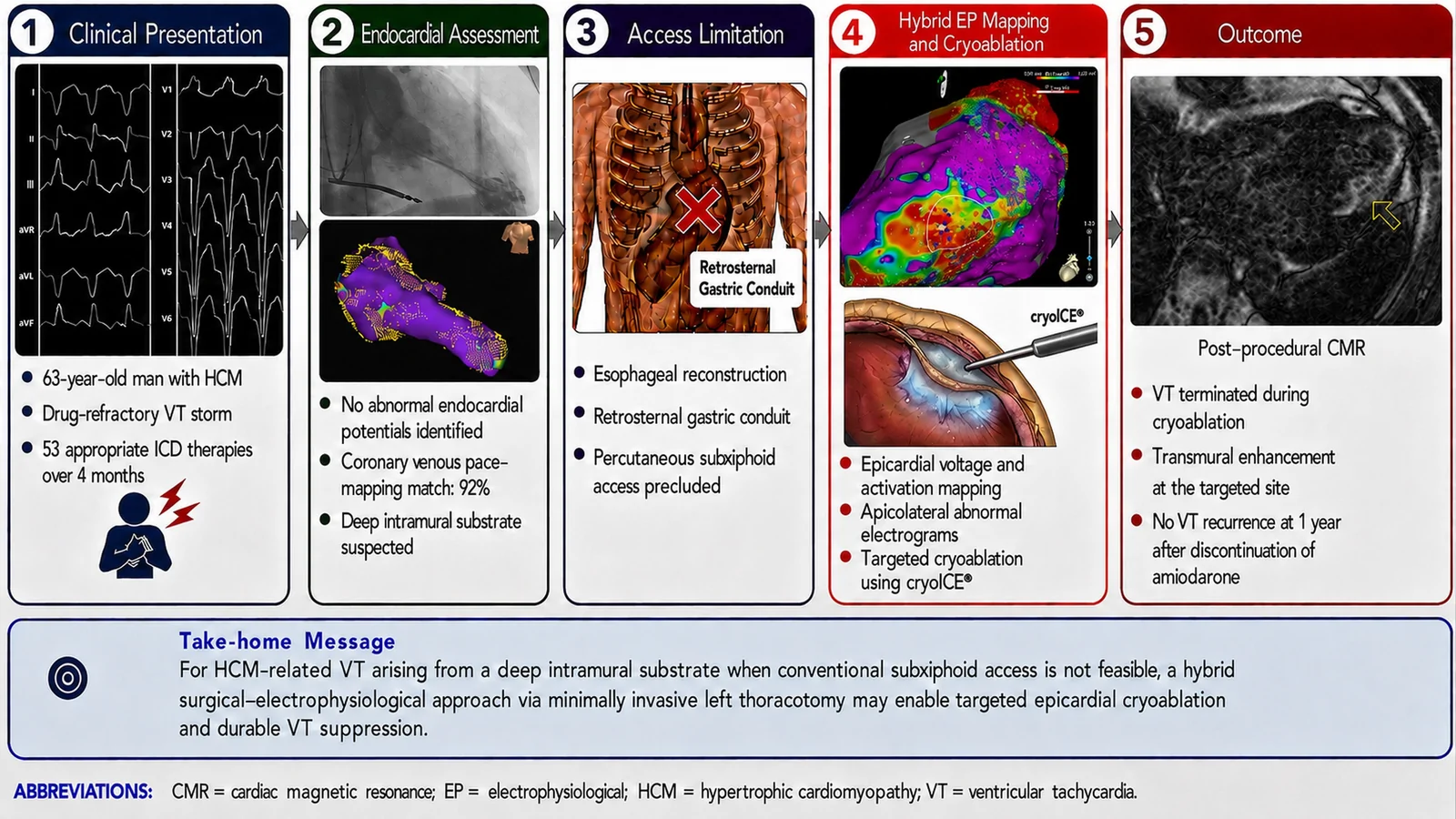


## Case presentation

A 63-year-old man with HCM presented with recurrent drug-refractory VT storm. There was no family history of HCM or sudden cardiac death, and genetic testing was not performed. Echocardiography showed marked midventricular obstructive HCM without a true apical aneurysm or left ventricular (LV) outflow tract obstruction. LV ejection fraction was 62%, with midventricular septal and lateral wall thicknesses of 15.3 and 14.3 mm, respectively. CMR performed in 2017 before implantable cardioverter-defibrillator (ICD) implantation showed late gadolinium enhancement (LGE) from the midventricular to apical myocardium. His medications included amiodarone 200 mg/day and bisoprolol 5 mg/day. Neither mavacamten nor aficamten had been administered. Despite treatment, the ICD delivered 53 appropriate therapies (45 antitachycardia pacing episodes and eight shocks) for VT over 4 months. During the first procedure, clinical VT was readily induced under general anaesthesia (*Figure 1Ai*). The LV was accessed via both retrograde transaortic and transseptal approaches. LV endocardial mapping using an HD Grid catheter (Abbott, Abbott Park, IL, USA) with the EnSite NavX system showed no abnormal potentials despite the narrowed mid-LV cavity (*Figure 1C* and *D*; Online Supplementary material online, *Video S1*). Endocardial radiofrequency ablation had no effect, and entrainment did not support endocardial involvement in the critical circuit. An EPstar Fix AIV 2.7-Fr catheter (Japan Lifeline, Tokyo, Japan) was positioned in multiple coronary venous branches for pace mapping. The best match (92%) was obtained in the distal anterolateral branch (*Figure 1Aii* and *B*), supporting a deeper intramural origin. Preprocedural computed tomography (CT) showed that the retrosternal gastric conduit created after oesophagectomy lay along the anticipated subxiphoid access route, rendering conventional percutaneous epicardial access unsafe. Therefore, the second procedure was performed using minimally invasive cardiac surgery (MICS) via left thoracotomy in a hybrid operating room, where the CARTO system was installed for intraoperative epicardial mapping. No significant pericardial adhesions were encountered. A limited pericardiotomy was created, and the OCTARAY catheter (Biosense Webster, Johnson & Johnson) was introduced into the pericardial space. To maintain stable three-dimensional localization, epicardial mapping was focused on the suspected apicolateral substrate. Clinical VT was readily induced by programmed ventricular stimulation. Epicardial voltage and activation mapping during VT identified a broad low-voltage area with fractionated and continuous electrograms at the apicolateral LV (*Figure 2*). Within the mapped region, the activation sequence accounted for only 46% of the VT cycle length. Together with the absence of a convincing endocardial target and the coronary venous findings, this was consistent with an epicardial breakout from a deeper intramural circuit. Epicardial cryoablation was performed using the cryoICE system (AtriCure, Cincinnati, OH, USA; Online Supplementary material online, *Video S2*). Given the parietal course of the left phrenic nerve, phrenic nerve injury was considered unlikely; nevertheless, care was taken during probe application. The epicardial coronary arteries were directly visualized and avoided. VT terminated 2.5 min after initiation of the first 5-min cryoapplication. Two additional 5-min applications were delivered at adjacent sites for lesion consolidation, with ventricular pacing at 90 bpm maintained after VT termination to prevent localized blood stasis and freezing. Epicardial fat was not removed; under direct visualization, perpendicular manual pressure was applied to maintain firm and stable cryoprobe contact with the epicardial surface. The prolonged applications were based on our preprocedural porcine experiments demonstrating time-dependent increases in lesion depth. After cryoablation, clinical VT was no longer inducible despite repeated programmed ventricular stimulation. No VT recurrence occurred during 1 year of follow-up after discontinuation of amiodarone, with only low-dose beta-blocker therapy. CMR demonstrated transmural enhancement at the ablation target site despite a myocardial thickness of approximately 15 mm (*Figure 3*).

**Figure 1:**
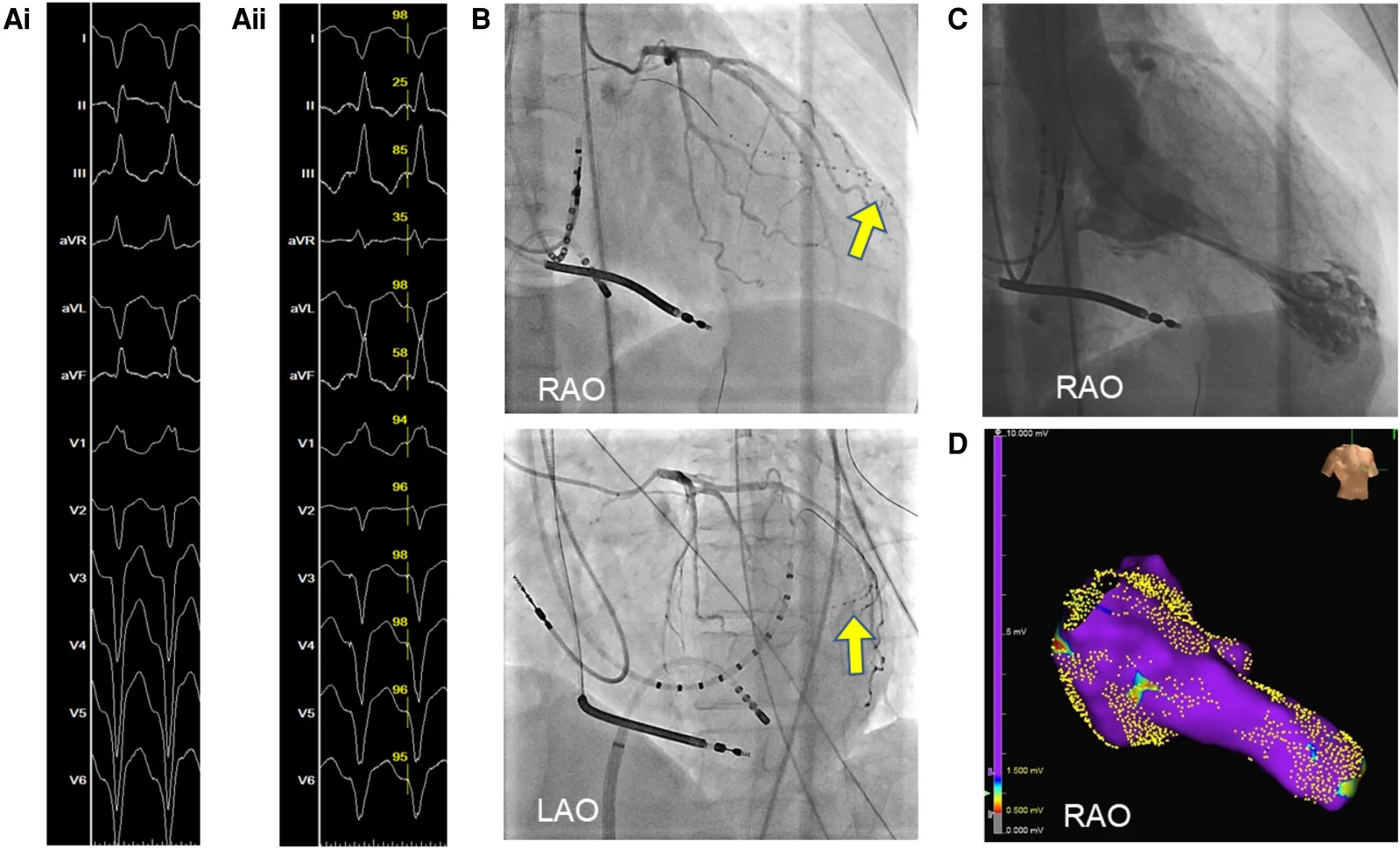
Electrocardiogram and multiple cardiac images. (*Ai*) Twelve-lead ECG of clinical VT. (*Aii*) Twelve-lead ECG during pace mapping at the distal anterolateral coronary venous branch, showing a 92% match. (*B*) Fluoroscopic images showing the EPstar Fix AIV 2.7-Fr catheter at the optimal pacing site in the distal anterolateral coronary venous branch in the RAO 30° and LAO 50° projections. (*C*) LVG demonstrating marked narrowing of the mid-LV cavity (see Online Supplementary material online, *Video S1*). (*D*) Endocardial bipolar voltage map of the LV obtained using an HD Grid catheter with the EnSite NavX system (voltage range, 0.5–1.5 mV), demonstrating the absence of abnormal endocardial potentials. ECG, electrocardiogram; LAO, left anterior oblique; LV, left ventricle; LVG, left ventriculography; RAO, right anterior oblique; VT, ventricular tachycardia.

**Figure 2:**
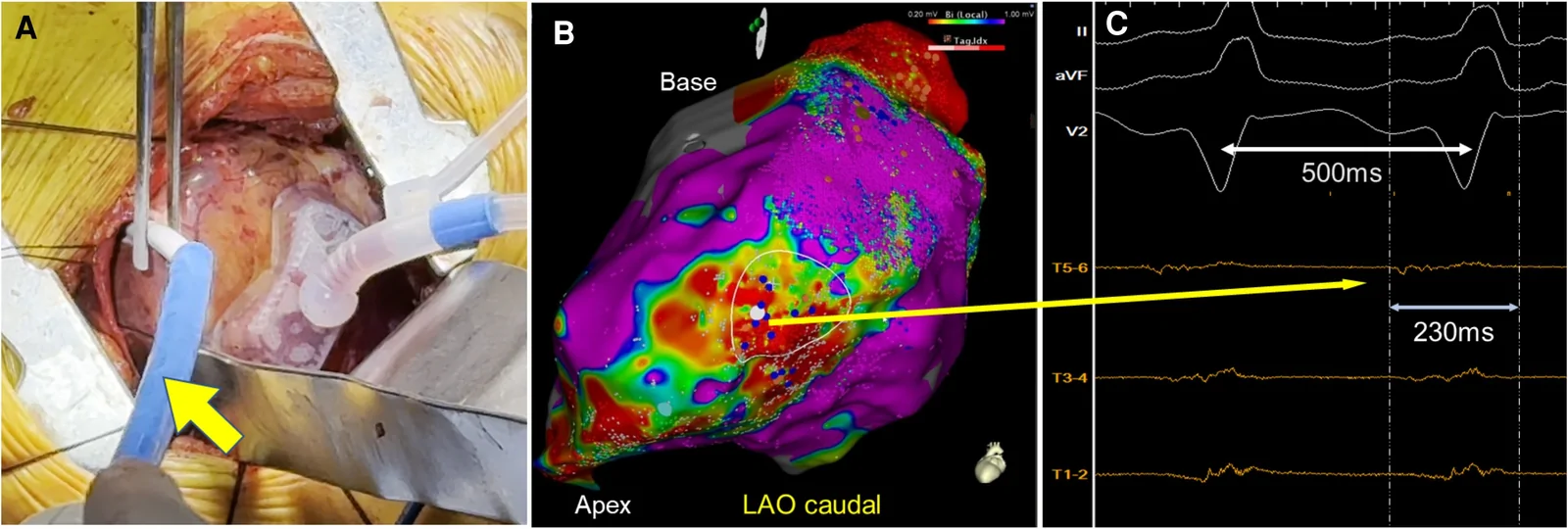
Epicardial cryoablation, three-dimensional mapping, and local electrograms. (*A*) Minimally invasive epicardial cryoablation via left thoracotomy using the cryoICE system (see Online Supplementary material online, *Video S2*) with a flexible, shape-adjustable aluminium tip (yellow arrow, a malleable cryotip with a 4-mm diameter and a 10-cm freeze length). (*B*) Epicardial bipolar voltage map of the LV obtained using an OCTARAY catheter with the CARTO 3 system (voltage range, 0.2–1.0 mV). Mapping was focused on the apicolateral LV surrounding the suspected VT substrate and demonstrated a broad low-voltage area. (*C*) Enlarged epicardial electrograms showing fractionated and continuous activity spanning 230 ms, corresponding to 46% of the 500-ms VT cycle length. Abbreviations as in Figure 1.

**Figure 3:**
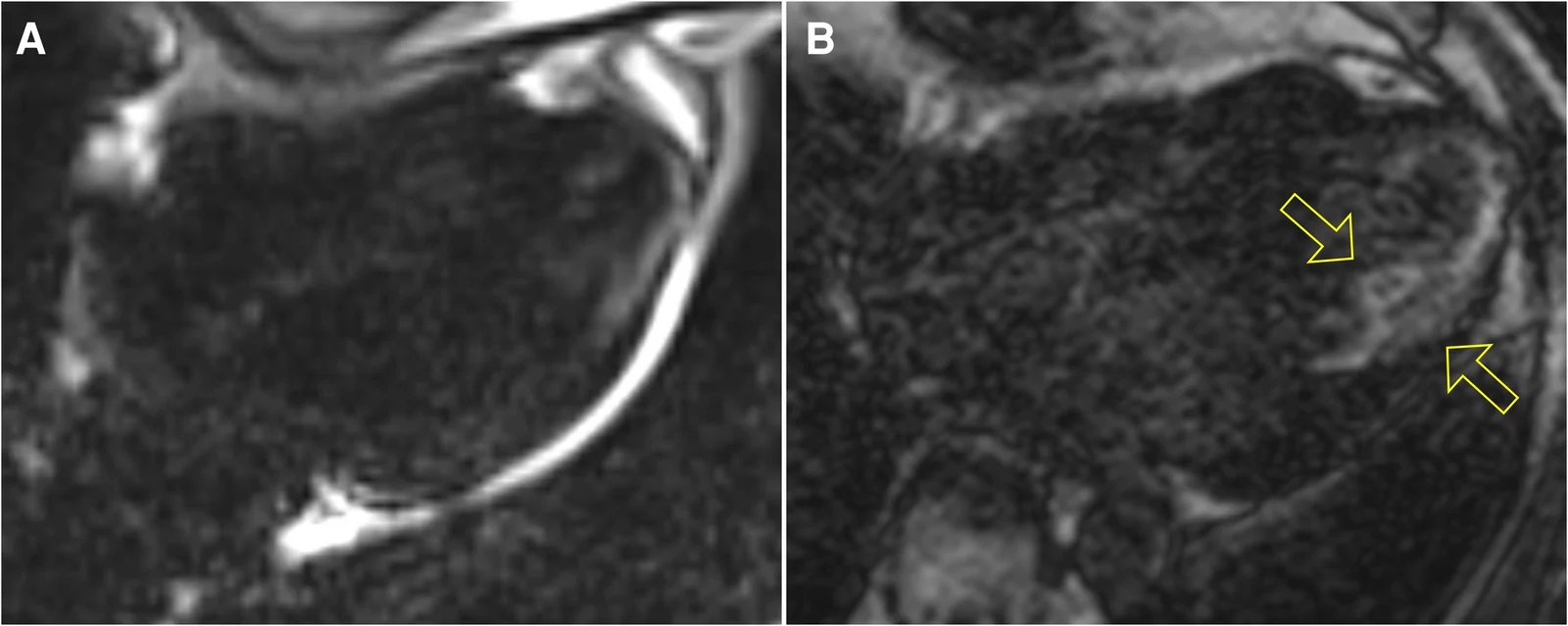
Pre- and postprocedural CMR findings. (*A*) Preprocedural 1.5-T LGE CMR image acquired in 2017 before ablation and ICD implantation. (*B*) Postprocedural 1.5-T LGE CMR image acquired in 2025 after ablation and in the presence of an ICD, demonstrating localized transmural enhancement at the apicolateral cryoablation target region (yellow arrows). Although both images were acquired at 1.5 T using LGE imaging and the most closely corresponding available anatomical levels were selected, direct image-to-image comparison remains limited by differences in acquisition sequences and image quality, including the use of an ICD-specific artefact-reduction technique for the post-procedural examination. CMR, cardiac magnetic resonance; ICD, implantable cardioverter-defibrillator; LGE, late gadolinium enhancement.

## Discussion

In the present case, LGE from the midventricular to apical myocardium and the absence of corresponding endocardial abnormalities suggested a deep intramural VT substrate. VT associated with HCM frequently originates from complex three-dimensional substrates involving intramural fibrosis and myocardial disarray. Previous studies have demonstrated that VT in HCM often arises from deep intramural circuits and is associated with lower ablation success rates than ischaemic VT because the critical isthmus may be located beyond the reach of conventional endocardial ablation techniques.^3,4^

A second important feature of this case was the inability to perform a conventional percutaneous epicardial approach. Previous retrosternal gastric conduit reconstruction created a substantial risk for subxiphoid pericardial access and necessitated an alternative strategy. Under these circumstances, minimally invasive left thoracotomy provided safe surgical access to the epicardial surface and enabled combined electrophysiological mapping and targeted ablation. Epicardial mapping identified fractionated and continuous electrograms within a low-voltage area at the apicolateral LV. Because epicardial mapping was focused on the suspected substrate, activation coverage was limited. Nevertheless, together with the absence of an endocardial target and the coronary venous pace-mapping findings, these findings were consistent with an epicardial breakout from a deeper intramural circuit.

Cryothermal energy was selected because it allows stable tissue contact and the creation of broad, homogeneous lesions under direct surgical visualization. Cryoablation produces sharply demarcated lesions with preservation of tissue architecture and progressive replacement fibrosis, characteristics that may facilitate the creation of deep and durable lesions in hypertrophied myocardium.^5^ Surgical cryoablation has previously demonstrated durable lesion formation and favourable long-term arrhythmia control in selected VT substrates.^6^ Despite an estimated myocardial thickness of approximately 15 mm, postprocedural CMR demonstrated localized transmural enhancement at the targeted cryoablation site, suggesting deep lesion formation. Preprocedural porcine experiments using the same cryoICE system demonstrated increasing lesion depth with longer single epicardial cryoapplications; repeated applications at the same site were not evaluated (see Supplementary material online, *Figure S1*).

Previous reports have described combined endocardial and epicardial ablation strategies for VT in HCM, including contemporary hybrid surgical approaches.^7^ However, epicardial access is occasionally impossible because of prior thoracic surgery, mediastinal reconstruction, or dense adhesions. In such situations, therapeutic options become extremely limited. The present case demonstrates that MICS can provide safe access to the epicardial surface and facilitate detailed electrophysiological mapping when conventional percutaneous access is not feasible.

This case illustrates that a hybrid surgical-electrophysiological strategy may represent an effective treatment option for patients with drug-refractory VT storm arising from deep intramural substrates when conventional epicardial access is not feasible.

A limitation of this report is that pathological confirmation of the intramural substrate was not available. Epicardial mapping was also limited to the region surrounding the suspected substrate rather than the entire ventricular epicardial surface. In addition, lesion depth was inferred from postprocedural CMR findings and experimental observations rather than direct histological assessment. Therefore, the mechanism of VT suppression should be interpreted cautiously. The closest available pre- and post-procedural 1.5-T LGE images were acquired using different sequences because ICD-specific artefact reduction was required postprocedurally, limiting direct comparison.

## Supplementary Material

ytag810_Supplementary_Data

## Data Availability

The data underlying this article are available from the corresponding author upon reasonable request.

## Lead author biography

Dr Tadashi Fujino received his MD degree from Toho University School of Medicine, Tokyo, Japan. He is currently Clinical Professor and electrophysiologist at the Arrhythmia Center, Toho University Omori Medical Center. His clinical and research interests include catheter ablation for atrial and ventricular arrhythmias, cardiac implantable electronic devices, and the prevention of sudden cardiac death.
