# Supplementary material for: Hybrid surgical-electrophysiological epicardial cryoablation for ventricular tachycardia storm from a deep intramural substrate in hypertrophic cardiomyopathy: a case report
Source: Eur Heart J Case Rep. 2026 Oct 27;10(11):ytag810. doi: 10.1093/ehjcr/ytag810 (PMC13760099; doi:10.1093/ehjcr/ytag810)
Supplement: Supplementary file 1 [file ytag810_supplementary_data.zip › Figure S1 legend.docx]

Figure S1: Each lesion wa s created by a single cryoapplication. Repeated cryoapplications at the same site were not evaluated in the porcine model.
